# Supplementary material for: Prevalence of mastitis and its associated risk factors in lactating camels in Northern Egypt
Source: PLoS One. 2025 Oct 7;20(10):e0333826. doi: 10.1371/journal.pone.0333826 (PMC12503299; doi:10.1371/journal.pone.0333826)
Supplement: Attachment — Submitted filename: Review plose.docx [file pone.0333826.s002.docx]

This research article, titled "Prevalence of Mastitis and Its Associated Risk Factors in Lactating Camels in Northern Egypt," published in PLOS ONE, presents a cross-sectional epidemiological investigation into camel mastitis across three governorates: Kafr El-Sheikh, Qalyubia, and Menofia. The study assessed 390 lactating camels using the California Mastitis Test (CMT) and identified both clinical and subclinical cases. The overall prevalence was 38.46%, with clinical mastitis accounting for 6.4% and subclinical mastitis for 32.1% of the examined animals. Multivariable logistic regression identified several significant risk factors: camels older than five years were 6.2 times more likely to be affected; early lactation stage increased risk by 2.5 times; tick infestation (OR=1.5), poor milking hygiene (OR=2.4), and the presence of udder or teat lesions (OR=2.5) were also strongly associated with higher mastitis prevalence. In contrast, locality and body condition score showed no significant association. The study’s methodology included clinical examination, milk sampling, and structured questionnaires, with statistical analyses performed using SPSS. The authors emphasize the economic and public health burden of subclinical mastitis, which frequently escapes detection, and advocate for improved management practices, including hygiene protocols and tick control measures. The study received ethical approval from Benha University, declares no funding or competing interests, and confirms that data are available upon reasonable request. Overall, this research provides important epidemiological data that can inform targeted interventions to improve udder health and milk safety in Egyptian camel populations.

**Review Comments and Suggestions**

- Kindly present the prevalence of clinical and subclinical mastitis at both the camel level and the quarter level separately.
- Table 2:The title of column 2 suggests that dogs were sampled - kindly correct this.
- Line 158: Kindly italicise Camelus dromedarius.

=
