# Supplementary material for: Prevalence of mastitis and its associated risk factors in lactating camels in Northern Egypt
Source: PLoS One. 2025 Oct 7;20(10):e0333826. doi: 10.1371/journal.pone.0333826 (PMC12503299; doi:10.1371/journal.pone.0333826)
Supplement: Attachment — Submitted filename: Reviewers comments on D-25-32223.docx [file pone.0333826.s003.docx]

**Comments on the manuscript entitled “Prevalence of Mastitis and Its Associated Risk Factors in Lactating Camels in Northern Egypt”**

**General Comments**

This is an informative study, revealing various risk factors for sub-clinical and clinical mastitis in camel. However, a significant weakness is the use of CMT as sole tool for mastitis diagnosis. For accurate and sensitive subclinical mastitis diagnosis, use of somatic cell count and/or isolation of pathogenic bacteria from milk are also suggested to include.

The manuscript is well written and statistical analysis is also appropriate.

**Materials & Methods**

The sub-clinical and clinical mastitis in camel was diagnosed using CMT that was originally developed for use in cattle. Though an old technique, it still serves as backbone of mastitis diagnosis. However, several paper has suggested certain modifications for improving diagnostic sensitivity and specificity in camel, as chemical properties of normal as well as mastitic camel milk differ from those in cattle. Authors should describe in detail the scoring system. I am unable to conclude what is 3% CMT reagent?

Furthermore, I am unable to decide whether the prevalence is calculated as animal basis or udder-quarter basis. The authors should clarify how many quarters were examined and how much were found positive for mastitis? Results should be presented as both quarter wise and animal wise prevalence.

Criteria for body condition scoring is not described.

**Specific Comments**

**Line No. 39**: Revise the sentence “It also contains higher phosphorus levels than other cattle”.
