# Supplementary material for: Prevalence of mastitis and its associated risk factors in lactating camels in Northern Egypt
Source: PLoS One. 2025 Oct 7;20(10):e0333826. doi: 10.1371/journal.pone.0333826 (PMC12503299; doi:10.1371/journal.pone.0333826)
Supplement: Attachment — Submitted filename: Response to reveiewe comments.doc [file pone.0333826.s005.doc]

**Comments to the Author**

1. Is the manuscript technically sound, and do the data support the conclusions?
The manuscript must describe a technically sound piece of scientific research with data that supports the conclusions. Experiments must have been conducted rigorously, with appropriate controls, replication, and sample sizes. The conclusions must be drawn appropriately based on the data presented.

Reviewer #1: Yes

Reviewer #2: No

2. Has the statistical analysis been performed appropriately and rigorously?

Reviewer #1: Yes

Reviewer #2: Yes

3. Have the authors made all data underlying the findings in their manuscript fully available?

The [PLOS Data policy](http://track.editorialmanager.com/CL0/http:%2F%2Fwww.plosone.org%2Fstatic%2Fpolicies.action%23sharing/1/010f019857770925-ccbe2b49-0af6-4803-8f8c-373ac88012c7-000000/sE5D4iWNiaG30eqAI2yEX-vzHe6dW5YwXXcHp22F9dk=222) requires authors to make all data underlying the findings described in their manuscript fully available without restriction, with rare exception (please refer to the Data Availability Statement in the manuscript PDF file). The data should be provided as part of the manuscript or its supporting information, or deposited to a public repository. For example, in addition to summary statistics, the data points behind means, medians and variance measures should be available. If there are restrictions on publicly sharing data—e.g. participant privacy or use of data from a third party—those must be specified.

Reviewer #1: Yes

Reviewer #2: Yes

4. Is the manuscript presented in an intelligible fashion and written in standard English?

PLOS ONE does not copyedit accepted manuscripts, so the language in submitted articles must be clear, correct, and unambiguous. Any typographical or grammatical errors should be corrected at revision, so please note any specific errors here.

Reviewer #1: Yes

Reviewer #2: Yes

5. Review Comments to the Author

This research article, titled "Prevalence of Mastitis and Its Associated Risk Factors in Lactating Camels in Northern Egypt," published in PLOS ONE, presents a cross-sectional epidemiological investigation into camel mastitis across three governorates: Kafr El-Sheikh, Qalyubia, and Menofia. The study assessed 390 lactating camels using the California Mastitis Test (CMT) and identified both clinical and subclinical cases. The overall prevalence was 38.46%, with clinical mastitis accounting for 6.4% and subclinical mastitis for 32.1% of the examined animals. Multivariable logistic regression identified several significant risk factors: camels older than five years were 6.2 times more likely to be affected; early lactation stage increased risk by 2.5 times; tick infestation (OR=1.5), poor milking hygiene (OR=2.4), and the presence of udder or teat lesions (OR=2.5) were also strongly associated with higher mastitis prevalence. In contrast, locality and body condition score showed no significant association. The study’s methodology included clinical examination, milk sampling, and structured questionnaires, with statistical analyses performed using SPSS. The authors emphasize the economic and public health burden of subclinical mastitis, which frequently escapes detection, and advocate for improved management practices, including hygiene protocols and tick control measures. The study received ethical approval from Benha University, declares no funding or competing interests, and confirms that data are available upon reasonable request. Overall, this research provides important epidemiological data that can inform targeted interventions to improve udder health and milk safety in Egyptian camel populations.

**Review Comments and Suggestions**

Point#: Kindly present the prevalence of clinical and subclinical mastitis at both the camel level and the quarter level separately.

Response# it was clarified

Point#: Table 2:The title of column 2 suggests that dogs were sampled - kindly correct this.

Response# it corrected

Point#: Line 158: Kindly italicise Camelus dromedarius.

Response# it edited

Reviewer 2:

**Comments on the manuscript entitled “Prevalence of Mastitis and Its Associated Risk Factors in Lactating Camels in Northern Egypt”**

**General Comments**

This is an informative study, revealing various risk factors for sub-clinical and clinical mastitis in camel. However, a significant weakness is the use of CMT as sole tool for mastitis diagnosis

**Point#:** For accurate and sensitive subclinical mastitis diagnosis, use of somatic cell count and/or isolation of pathogenic bacteria from milk are also suggested to include.

The manuscript is well written and statistical analysis is also appropriate.

**Materials & Methods**

**Point#:** The sub-clinical and clinical mastitis in camel was diagnosed using CMT that was originally developed for use in cattle. Though an old technique, it still serves as backbone of mastitis diagnosis. However, several paper has suggested certain modifications for improving diagnostic sensitivity and specificity in camel, as chemical properties of normal as well as mastitic camel milk differ from those in cattle. Authors should describe in detail the scoring system. I am unable to conclude what is 3% CMT reagent?

**Point#:** Furthermore, I am unable to decide whether the prevalence is calculated as animal basis or udder-quarter basis. The authors should clarify how many quarters were examined and how much were found positive for mastitis? Results should be presented as both quarter wise and animal wise prevalence.

Response#: it was clarified and added

**Point#:** Criteria for body condition scoring is not described.

Response#: it added

**Specific Comments**

**Point#:** **Line No. 39**: Revise the sentence “It also contains higher phosphorus levels than other cattle”.

Response#: it deleted

=
