# Supplementary material for: Prevalence of mastitis and its associated risk factors in lactating camels in Northern Egypt
Source: PLoS One. 2025 Oct 7;20(10):e0333826. doi: 10.1371/journal.pone.0333826 (PMC12503299; doi:10.1371/journal.pone.0333826)
Supplement: Attachment — Submitted filename: response to reviewer comments.doc [file pone.0333826.s006.doc]

**Point#:** Table1 and table 2 are somewhat duplicated and should be compiled into a single table.

In such a table, you should indicate one column for « No of positive camels ^a^», with its associated « Camel-level prevalence %» column, and a one column for « No of positive quarter samples ^b^» and a « Quarter-level prevalence % » column. Subscripts a and b should indicate the total number of camels and quarters respectively as a footnote of the table.

Response#: both tables were merged

**Point#:** In table 5, you should specify the distribution between clinical and subclinical cases. For instance, for each pathogen, you could mention in parenthesis the number of clinical and subclinical cases from which this pathogen was identified. If, as an example, among the 95 E. coli isolates 20 are from subclinical casees, you could indicate 95 (20/75).

Response#: it rephrased

**Point#:** Furthermore, you should clarify how you selected the 600 milk samples for bacteriological analysis since you only observed 450 mastitic quarters. For this last comment, I insist that you not only correct the manuscript file but also give details in your response to this comments. You will have to adjust the abstract.

Response#: The results revealed that 150 animals tested positive. From these animals, 600 milk samples representing their quarters were subjected to bacteriological examination.
